# COVID-19 vaccine uptake and associated factors among individuals living in a peri-urban area in Uganda: A cross-sectional study

**DOI:** 10.1371/journal.pone.0312377

**Published:** 2024-11-04

**Authors:** Mary Bridget Nanteza, Gertrude Nanyonjo, Nasimu Kyakuwa, Flavia Nakanjako, Hamza Kalute, Christine Atuhairwe, Christine Watera, Deogratius Ssemwanga

**Affiliations:** 1 Expanded Program on Immunization Laboratory, Uganda Virus Research Institute (UVRI), Entebbe, Uganda; 2 UVRI-International AIDS Vaccine Initiative (UVRI-IAVI), Entebbe, Uganda; 3 Faculty of Health Sciences, Uganda Martyrs University, Kampala, Uganda; 4 Medical Research Council /UVRI and London School of Hygiene and Tropical Medicine, Entebbe, Uganda; University of Environment and Sustainable Development, GHANA

## Abstract

**Introduction:**

The Corona virus disease (COVID-19) is a respiratory illness that is caused by SARS-CoV-2 virus. This virus was first reported in China in December 2019. It then spread to all countries and from March 11, 2020, the World Health Organization declared the COVID-19 outbreak a pandemic. In Uganda, the disease was first reported in March 2020 and COVID-19 vaccines became available by January 2021. Although COVID-19 vaccines were available in Uganda, uptake remained low. The aim of this study was to establish COVID-19 vaccine uptake awareness in a peri-urban setting in Entebbe City, Uganda.

**Methods:**

This was a cross-sectional study conducted among 127 men and 263 women who reside in Entebbe City, Uganda. Data was collected on socio-behavioral characteristics, knowledge, attitude, and practice (KAP) about COVID-19 vaccine using interviewer administered questionnaires. Uptake of COVID-19 vaccine was defined as the proportion of participants who had received at least one dose of the COVID-19 vaccine. We used descriptive statistics to estimate awareness of COVID-19 vaccines. The ‘chi-square test’ and ‘modified Poisson regression’ were used to assess variations in uptake of COVID-19 vaccines among respondents and their socio-demographics as well as other characteristics.

**Results:**

Ninety-nine percent (388 out of 390) of the study population were aware of at least one brand of COVID-19 vaccines in the country. Thirty-five percent (138 out of 390) knew that the vaccine immunity was achieved 14 days after the 2^nd^ dose and 98.7% (385 out of 390) admitted that observing the standard operating procedure for COVID-19 infection prevention was necessary after vaccination. There was a gap in knowledge on vaccine safety reported by 74.6% (291 out of 390) participants. Some participants 37.2% (145 out of 390) had concerns about the vaccine. Of these, 57.9% (84 out of 145) believed that the vaccines were not helpful; and 30.3% (44 out of 145) feared serious side effects. Sixty-six percent (257 out of 390) believed that vaccines were not working and 79.0% (308 out of 390) admitted that vaccines were promoted for financial gain. At the time of performing the study, 36.2% and 22.3% had received the 1^st^ and 2^nd^ dose respectively. The main sources of information on COVID-19 vaccine were television (TV) and social media (p-value 0.001). In a multivariate model, COVID-19 vaccine acceptability was associated with salaried and self-employment (p-value 0.046). The other predicative factors were awareness of the COVID-19 vaccine (p-value <0.001) and having vaccine concerns (p-value 0.013).

**Conclusion:**

Uptake of COVID-19 vaccination in Entebbe community was low, partly attributed to knowledge gaps and concerns about vaccine safety and effectiveness. This highlights the need to enhance dissemination of information about COVID-19 vaccine. The lessons learnt in this study would be relevant for other emerging infections by informing vaccination implementation programs in similar settings.

## Introduction

SARS COV-2 transmission and the fulminating COVID-19 disease can be prevented by SARS-COV-2 vaccines that became globally available in December 2020 [1–3]. In 2021, a cross-sectional study in Africa showed vaccine acceptance levels of 48.93% (95% CI [39.49, 58.37] [4]. However, in April 2022, vaccine coverage of only 12% was reported for Eastern and Southern Africa [5]. By September 2022, the vaccine uptake in Africa was 22.2% still below the WHO target of 70% of the total population who had been fully immunised [6]. As of December 2022, COVID-19 vaccine coverage barely increased and was estimated at 26.4% [7]. Overall, vaccination success has been hindered by negative perception about the COVID-19 disease and vaccines [8–10]; in addition to the absence of vaccines which was initially a challenge for Africa. The situation in Africa poses a challenge to the success of future vaccination programmes [11, 12].

In Uganda, the government secured COVID-19 vaccines with the support from UNICEF and GAVI Alliance [13, 14]. Vaccination began in March 2021. Despite availability of free vaccines, vaccination uptake in Uganda remained low. It was rated at 40–50% for those who received the 1^st^ dose and 30–40% for those who received the 2^nd^ dose as of December 2022 [15]. From a recent study, vaccine uptake that was observed in estate and slum communities in Uganda was reported at 43.8% and 39.9% respectively [16]. These communities were in Kampala and Wakiso districts; the slum and estate communities were not specified. Another study performed in a rural setting of Uganda described low education level and misconceptions about the disease and vaccines as obstacles to vaccine uptake [10]. The low incidence of COVID-19 disease in the country was also associated with poor compliance to vaccine uptake [17, 18]. Others believed in divine intervention for a break-through [19]; and persons from malaria endemic areas were believed to be protected from the virus [20, 21]. A study conducted among Ugandan women reported an erroneous belief that COVID-19 infection was believed to be a disease of the white race manifesting with high morbidity and mortality [22]. Uptake of COVID-19 vaccine was also reported to be affected by the level of civilization, societal factors and age [23–25]. Contrary, studies performed among the literate Ugandans aged >18 years displayed good attitudes and prevention approaches estimated at 72.4% and 85.3% respectively [26]. Numerous factors have been shown to contribute to the low COVID-19 vaccine uptake in Uganda. This study aimed to determine COVID-19 vaccine uptake awareness and the associated positive and negative factors that could be promoted or discouraged to enhance vaccine uptake in the country. The study was conducted at a clinic in the peri-urban area because it was a catchment site in the proximity of the investigating institution, Uganda Virus Research Institution (UVRI).

## Materials and methods

### Study design

This was a cross-sectional study conducted among 390 individuals (263 female) between 10^th^ November 2021 and 2^nd^ February 2022. An interviewer administered questionnaire was used to collect data on socio-behavioral characteristics, knowledge, attitude, and practice (KAP) about COVID-19 vaccine.

### Sampling procedure

Selection of respondents was by convenience sampling. All people who came to the clinic during the study period were given information about the study. Those who fulfilled the eligibility criteria and were willing to participate in the study were recruited.

### Study setting

The study was conducted in peri-urban communities of Entebbe City in Uganda. Entebbe is a small city of around 70,000 people located on the shores of Lake Victoria, 40 km south of Kampala, the capital city of Uganda. The study included two main communities Katabi town council and Entebbe Central division. Selection of participants was open to those who came to seek health services and vaccination at the UVRI Clinic.

### Data collection procedures

Individuals who visited the clinic were briefed about the study and those who were interested in participating were grouped and given further details about the study. After obtaining informed consent, they were asked to respond to a set of questions administered by trained research assistants. Each participant was interviewed for 30 to 40 minutes. The recruitment exercise started on 10^th^ November 2021 and was concluded on 2^nd^ February 2022.

### Description and conceptualization of variables

The independent variables for knowledge included whether the participant had ever heard about COVID-19 vaccines, and the source of information. The independent variables for practices included willingness to take COVID-19 vaccines, willingness to pay for the vaccines, and whether they would recommend the vaccine to a friend or family. The independent variables for attitude included having received adequate information on the COVID-19 vaccine safety, satisfaction with the health professional/health worker’s responses on COVID-19 vaccine, having concerns about the COVID-19 vaccine, taking precautions after vaccination, requirement for other information/messages on the coronavirus, and the level of trust of COVID-19 information. Finally, the independent variables for perceptions included concerns about the efficacy of the vaccines, and whether taking precaution after vaccination was still important and whether one needed more information.

### Study variables

Our outcome variable was uptake of any type of COVID-19 vaccine measured basing on whether the study participant managed to receive the COVID-19 vaccine using a binary scale of yes or no. We defined COVID-19 vaccine uptake as the proportion of participants who had received at least one dose of any COVID-19 vaccine at the time of performing the study.

The independent variables included socio-demographics such as age (measured in years); sex (male and female); marital status (married/cohabiting, single, separated/divorced, widowed); level of education completed (none, primary, secondary, tertiary/vocational); religious affiliation (Christian, Moslem); and occupation (formal-also described as salaried, both salaried and self-employed, informal, self-employed).

Trusted sources (newspapers, social media, radio, internet or church); trusted information about COVID-19 vaccines (very trusted, moderately, somewhat, not trusted); mandatory vaccination (no or yes); trusted that the vaccine protects against COVID-19 disease (disagree, agree, neutral); immunity against corona virus achieved (after first dose, second dose, 14 days after first dose, 14 days after second dose, or don’t know); vaccine eligibility (no or yes); source of COVID-19 vaccine information (doctor, nurse, social media, radios, television shows); practices for COVID-19 vaccine uptake (willingness to take, pay, and recommend vaccination to others); motivation, and hindering factors. The key questions were borrowed from a validated questionnaire [27]. These have been provided in S1 Appendix.

### Quantitative data

We double-entered quantitative data using Epi-Data version 3.1 and merged the files to ensure precision, concurrently ensuring quality control measures and then exported the data to Stata version 15 for analysis.

### Statistical methods

In the first stage of analysis, descriptive statistics were used to estimate uptake of COVID-19 vaccines. The chi-square test was used to compare the uptake of COVID-19 vaccines of the study participants with their socio-demographics and other characteristics. For variables with an expected cell frequency count less than 5, we reported the Fisher’s exact test p-value but not the p-value for the Chi-square test. Variables found significant at p≤0.05 in the bivariate analyses were further modelled into the modified Poisson regression analysis to determine those independently associated with the outcome.

### Ethical approval

The study was approved by the Uganda Virus Research Institute, Research and Ethics Committee (UVRI REC) Reference GC/127840 and the Uganda National Council for Science and Technology (UNCST) Reference HS1660ES. Participants were given detailed information about the study through an information sheet read to them in the language they understood best that was either English or Luganda. Documented informed consent was sought from all study participants. The participants were reimbursed $ 3.00 for their time and transport.

## Results

A total of 390 participants consented and were included in the analysis. Of these 67.4% were females. The median age was 26 years [interquartile range (IQR) 21–32] and the majority were aged 21–30 years. Half (51.0%) were married, 63.3% had attained secondary education, 89.7% were Christians and 52.8% were self-employed. Ninety-nine percent (99.5%) had heard of the COVID-19 vaccine, of whom 34.9% and 27.2% heard it from television (TV) shows and social media respectively. Table 1 below shows more details on the socio-demographic characteristics.

**Table 1:** Socio-demographic characteristics of the study participants.

Characteristics	Level	N = 390(%)
Gender	Male	127(32.6%)
	Female	263(67.4%)
Age group		
	Less than 20 Years	73(18.7%)
	21–30 Years	199(51.0%)
	31–40 Years	72(18.5%)
	More than 41 Years	46(11.8%)
Marital status		
	Married	199(51.0%)
	Single/never married	20(5.1%)
	Separated/divorced	163(41.8%)
	Widowed	8(2.1%)
Level of education		
	None	7(1.8%)
	Primary	73(18.7%)
	Secondary	247(63.3%)
	Tertiary/vocational	63(16.2%)
Religious affiliation		
	Christian	350(89.7%)
	Moslem	40(10.3%)
Occupation		
	Formal (salaried)	59(15.1%)
	Both salaried & self employed	71(18.2%)
	Informal	54(13.9%)
	Self-employed	206(52.8%)
Heard of COVID-19 vaccine		
	No	2(0.5%)
	Yes	388(99.5%)
Source of COVID-19 vaccine knowledge		
	Doctor	70(17.9%)
	Nurse	6(1.5%)
	Social media	106(27.2%)
	Radio	31(7.9%)
	TV shows	136(34.9%)
	Others	41(10.5%)

Overall, 74.6% of the study participants reported that they had not received adequate information on COVID-19 vaccine safety. Approximately thirty-seven percent, 37.7%, had concerns about the COVID-19 vaccines, 30.3% of whom had fears of the side effects and 57.9% believed the vaccines were not helpful. Interestingly, 98.7% admitted that observing standard operating procedures for COVID-19 infection prevention was still necessary irrespective of having received the vaccine. Only 36.2% and 22.3% had received the 1^st^ and 2^nd^ dose of the vaccine respectively. Thirty-five (35.4%) reported that the immunity of COVID-19 vaccine was achieved 14 days after the 2^nd^ dose. Table 2 shows additional information on the knowledge, attitudes, and perceptions on the vaccine.

**Table 2:** Knowledge, attitude, perception and concerns about the COVID-19 vaccine.

Characteristics	Level	All (n = 390) n(%)
Felt that they received adequate information on the COVID-19 vaccine safety		
	Disagree	291(74.6%)
	Neutral	8(2.1%)
	Agree	91(23.3%)
Satisfied with the health professional/health worker’s responses on COVID-19 vaccine		
	Disagree	349(89.5%)
	Neutral	11(2.8%)
	Agree	30(7.7%)
Had concerns on the COVID-19 vaccine		
	No	243(62.3%)
	Yes	147(37.7%)
*Main concern about the COVID-19 vaccines*		
	*Already healthy*	*1(*.*7%)*
	*Fear of injection pain*	*14(9*.*7%)*
	*Side effects of vaccines*	*44(30*.*3%)*
	*Vaccines not helpful*	*84(57*.*9%)*
	*Others*	*2(1*.*4%)*
Taking precaution after vaccination		
	Still important	385(98.7%)
	Less important	1(0.3%)
	Not important	4(1.0%)
Would like other information/messages on the coronavirus		
	No	173(44.4%)
	Yes	217(55.6%)
Took the COVID-19 vaccines		
	No	162(41.5%)
	Yes (First dose)	141(36.2%)
	Yes (Both doses)	87(22.3%)
Trust level of COVID-19 information		
	Very trusted	293(75.1%)
	Moderately trusted	25(6.4%0
	Somewhat trusted	56(14.4%)
	Not trusted at all	16(4.1%)
Immunity against COVID-19 is achieved after		
	First dose	51(13.1%)
	Second dose	75(19.2%)
	14 Days after first dose	43(11.0%)
	14 Days after second dose	138(35.4%)
	Don’t know	83(21.3%)

Fifty-two percent of the study participants knew the vaccine eligibility status for patients with chronic infection and those with immune suppression states (52.6% and 73.6%). Regarding the significance or impact of the vaccine information source, social media ranked high with 68.7% and the impact of religious leaders was low with 24.9%. News from the national radio/TV ranked least at 8.5%. When participants were asked about their willingness to accept the COVID-19 vaccine, majority (95.9%) were unwilling with only 9.5% willing to pay for the vaccine. Unforeseen future effects of COVID-19 vaccine were a concern for 60.0% of participants. Table 3 below elaborates on the practices and concern for COVID-19 vaccine.

**Table 3:** Practices and concerns regarding the COVID-19 vaccine.

Characteristics	Level	n (%)
**Groups eligibility of COVID-19 vaccines**		
Patients with chronic diseases		
	Eligible	205(52.6%)
	Not eligible	138(35.4%)
	Don’t know	47(12.1%)
Persons allergic to food items/drugs		
	Eligible	252(64.6%)
	Not eligible	22(5.6%)
	Don’t know	9(2.3%)
Immune compromised patients		
	Eligible	287(73.6%)
	Not eligible	73(18.7%)
	Don’t know	30(7.7%)
**The sources of information and their significance**		
News from national TV/Radio	Insignificant effect	
	Somewhat significant	327(83.8%)
	Very significant	30(7.7%)
		33(8.5%)
Social media (Facebook, WhatsApp)		
	Insignificant effect	91(23.3%)
	Somewhat significant	31(7.9%)
	Very significant	268(68.7%)
Religious leader	Insignificant effect	258(66.2%)
	Somewhat significant	35(9.0%)
	Very significant	97(24.9%)
**Practices for COVID-19 vaccine uptake**		
Willingness to take the COVID-19 vaccine		
	Disagree	374(95.9%)
	Neutral	4(1.0%)
	Agree	12(3.1%)
Willing to pay to get COVID-19 vaccine		
	Disagree	344(88.2%)
	Neutral	9(2.3%)
	Agree	37(9.5%)
Would recommend friends and family for vaccination	Disagree	379(97.2%)
	Neutral	1(.3%)
	Agree	10(2.6%)
**Concerns of COVID19 vaccines**		
Fear of serious side effects after vaccination	Disagree	178(45.6%)
	Neutral	9(2.3%)
	Agree	203(62.1%)
COVID-19 vaccine may be faulty or fake		
	Disagree	115(29.5%)
	Neutral	18(4.6%)
	Agree	257(65.9%)
Unforeseen future effects of COVID-19 vaccine		
	Disagree	137(35.1%)
	Neutral	19(4.9%)
	Agree	234(60.0%)
Vaccine promoted for commercial gain		
	Disagree	57(14.6%)
	Neutral	25(6.4%)
	Agree	308(79.0%)

Key: Strongly disagree/disagree, N-Neither disagree nor agree, A-Strongly agree/agree

At bivariate analysis, the socio-demographic factors associated with vaccine acceptability were age (p-value 0.041), occupation (p-value 0.003) and sources of COVID-19 vaccine information (p-value 0.001). Other socio-demographic variables were insignificant (Table 4).

**Table 4:** Bivariate analysis of COVID-19 vaccine acceptability by socio-demographic factors.

Characteristics	Accepted COVID-19 vaccine			
	No (n = 162)	Yes(n = 228)	All (n = 390)	p-value
**Gender**				
Male	52(40.9)	75(59.1)	127	0.869
Female	110(41.8)	153(58.2)	263	
**Age group**				
<20 Years	40(54.8)	33(45.2)	73	0.041*
21–30 Years	71(35.7)	128(64.3)	199	
31–40 Years	31(43.1)	41(56.9)	72	
>41 Years	20(43.5)	26(56.5)	46	
**Marital status**				
Married	80(40.2)	119(59.8)	199	0.797
Single	7(35.0)	13(65.0)	20	
Separated/divorced	72(44.2)	91(55.8)	163	
Widowed	3(37.5)	5(62.5)	8	
**Level of education**				
None	2(28.6)	5(71.4)	7	0.231
Primary	35(47.9)	38(52.1)	73	
Secondary	105(42.5)	142(57.5)	247	
Tertiary/vocational	20(31.7)	43(68.3)	63	
**Religious affiliation**				
Christian	144(41.1)	206(58.9)	350	0.639
Moslem	18(45.0)	22(55.0)	40	
**Occupation**				
Formal	25(42.4)	34(57.6)	59	0.003*
Both salaried & self employed	16(22.5)	55(77.5)	71	
Informal	23(42.6)	31(57.4)	54	
Self-employed	98(47.6)	108(52.4)	206	
**Heard of COVID-19 vaccine**				
No	1(50.0)	1(50.0)	2	0.808
Yes	161(41.5)	227(58.5)	388	
**Source of COVID-19 vaccine knowledge**				
Doctor	20(28.6)	50(71.4)	70	0.001*
Nurse	1(16.7)	5(83.3)	6	
Social media	41(38.7)	65(61.3)	106	
Radio	7(22.6)	24(77.4)	31	
TV shows	68(50.0)	68(50.0)	136	
Others	25(61.0)	16(39.0)	41	

* p<0.05 statistically significant

The other factors associated with vaccine acceptability were participant’s concerns on the COVID-19 vaccine (p-value 0.006); trusted sources of COVID-19 information (p-value 0.023); and willingness to being vaccinated (p-value 0.003). Participants who had no concerns about the COVID-19 vaccine (63.8%) accepted the vaccine. The church contributed to the dissemination of COVID-19 vaccine information by 54.2%, and radio 61.2%. Fifty-seven percent (57.2%) of participants who had disagreed that the vaccines were protective against severe disease did accept the vaccine though the finding was not statistically significant. Many participants admitted that immunity to the COVID-19 vaccine was achieved 14 days after the second dose (60.1%) whereas 50.6% had no idea about it. Again, this was not statistically significant p-value 0.309. Surprisingly, unwillingness to take the vaccine did not result in refusal to take the vaccine (25.0%) compared to the 60.2% who agreed and took the vaccine p-value 0.003 (Table 5).

**Table 5:** Bivariate analysis of COVID-19 vaccine acceptability by knowledge, attitude, and concerns.

Characteristics	Accepted COVID-19 vaccine			
	No (n = 162)	Yes(n = 228)	All (n = 390)	p-value
**Had concerns on the COVID-19 vaccine**				
No	88(36.2)	155(63.8)	243	0.006*
Yes	74(50.3)	73(49.7)	147	
**Taking precaution after vaccination**				
Still important	160(41.6)	225(58.4)	385	0.661
Less important	0(0.0)	1(100.0)	1	
Not important	2(50.0)	2(50.0)	4	
**Would like more information on the coronavirus**				
No	66(38.2)	107(61.8)	173	0.225
Yes	96(44.2)	121(55.8)	217	
**Trusted sources of COVID-19 information**				
News papers	0(0.0)	7(100.0)	7	0.023*
Social media	2(15.4)	11(84.6)	13	
Radio	33(38.8)	52(61.2)	85	
Internet	12(35.3)	22(64.7)	34	
Church	115(45.8)	136(54.2)	251	
**Trust level of COVID-19 information**				
Very trusted	116(39.6)	177(60.4)	293	0.352
Moderately trusted	14(56.0)	11(44.0)	25	
Somewhat trusted	26(46.4)	30(53.6)	56	
Not trusted at all	6(37.5)	10(62.5)	16	
**Mandatory to take COVID-19 vaccine**				
No	51(44.7)	63(55.3)	114	0.410
Yes	111(40.2)	165(59.8)	276	
**Believe the COVID-19 protects from severe infection**				
Disagree	142(42.8)	190(57.2)	332	0.386
Neutral	4(26.7)	11(73.3)	15	
Agree	16(37.2)	27(62.8)	43	
**Immunity against COVID-19 is achieved after**				
First dose	21(41.2)	30(58.8)	51	0.309
Second dose	25(33.3)	50(66.7)	75	
14 Days after first dose	20(46.5)	23(53.5)	43	
14 Days after second dose	55(39.9)	83(60.1)	138	
Don’t know	41(49.4)	42(50.6)	83	
**Willingness to vaccinate**				
Disagree	9(75.0)	3(25.0)	12	**0.003**
Neutral	4(100.0)	0(0.0)	4	
Agree	149(39.8)	225(60.2)	374	

* p<0.05 statistically significant

In the multivariate model, the factors associated with COVID-19 vaccine acceptability were salaried and self-employment (cPR1.30; 95% CI:1.00–1.68, p-value 0.046), hearing of the COVID-19 vaccine (cPR0.914; 95% CI: 0.87–0.95, p-value <0.001), and having vaccine concerns (cPR 0.78; 95% CI: 0.65–0.95, p-value 0.013) (Table 6).

**Table 6:** Model summary.

Category	IRR.	p-value	[95% Conf	Interval]	Sig
**Gender**					
Male	1	.	.	.	
Female	0.958	0.643	0.799	1.149	
**Age Group**					
<20 Years	1	.	.	.	
21–30 Years	1.265	0.110	0.948	1.686	
31–40 Years	1.110	0.546	0.791	1.556	
>41 Years	1.085	0.676	0.739	1.594	
**Education**					
None	1	.	.	.	
Primary	0.776	0.264	0.498	1.211	
Secondary	0.807	0.309	0.534	1.22	
Tertiary/Vocational	0.926	0.733	0.595	1.441	
**Occupation**					
Formal (salaried)	1	.	.	.	
Both salaried & self employed	1.301	0.046	1.004	1.687	**
Informal	1.054	0.738	0.773	1.438	
Self-employed	0.946	0.666	0.734	1.219	
**Heard of COVID 19 vaccine**					
No	1	.	.	.	
Yes	0.914	<0.001	0.873	0.958	***
**Had Covid 19 vaccine concerns**					
No	1	.	.	.	
Yes	.789	.013	.655	.951	**
**Patients with chronic diseases**					
Eligible	1	.	.	.	
Not eligible	0.887	0.197	0.740	1.064	
Don’t know	1.059	0.659	0.820	1.369	
**Religious leaders**					
Insignificant	1	.	.	.	
Somewhat significance	1.273	0.051	0.999	1.622	*
Very significant	1.092	0.368	0.901	1.323	
**Willingness to be vaccinated**					
Disagree	1	.	.	.	
Neither agree nor disagree	4.74	.	1.18	0.001	
Agree	2.174	1.112	0.834	5.671	
**Willing to pay for the vaccine**					
Disagree	1	.	.	.	
Neither agree nor disagree	0.612	0.270	0.256	1.465	
Agree	1.068	0.664	0.794	1.437	
Constant	0.918	0.777	0.508	1.660	

* p<0.05 statistically significant

## Discussion

This study assessed COVID-19 vaccine uptake awareness in a peri-urban setting in Entebbe City, Uganda. In Entebbe community, 36.2% and 22.3% of the population received the 1^st^ and 2^nd^ dose of COVID-19 vaccine respectively from November 2021 to December 2021. By June 2022, the vaccine uptake of the first dose had increased to 42% [28]. However, this was below the WHO target for the first dose set at 70% for the eligible population. As of October 2023, 26,406,936 doses of the vaccine had been administered. These were inadequate for the ≥ 24.7 million eligible population considering the two doses recommended for most COVID-19 vaccines used in Uganda [29]. Due to a difference in the data computing for year 2023, it was not possible to track and monitor the vaccine coverage levels. Ninety-nine percent (99%) of the individuals were aware of at least one brand of COVID-19 vaccines.

From this study, the major hindrance to COVID-19 vaccine uptake was misconception about the vaccine (p-value 0.006), which has been a common finding in related studies [9, 10]. Misconceptions included beliefs that vaccines were not effective and were promoted for financial gain. In addition, many participants had a concern about the possible short-term and long-term side effects of the vaccines. Many participants did not know that COVID-19 vaccine protects from severe COVID-19 disease. The gaps observed in the setting should be addressed and communicated to the public using impactful information transfer platforms. Other modes of communication should also be promoted since dissemination tools target different population groups.

In this study, females were more willing to receive the vaccine. In Uganda, females reportedly have a better health seeking behavior than males [30]: females have a better opportunity to receive health education as they attend antenatal and immunization care clinics [31] and thus, are more empowered and would make positive decisions on health issues easily. However, in a systematic review and meta-analysis performed among studies conducted in high-income countries, men had a higher intention to receive the vaccines. The difference in acceptance was more observed among the health care workers compared to the general population [32]; thus, the difference observed could be linked to health promotion sensitization. In another study, gender difference did not influence the perception on COVID-19 vaccination [33]. As mentioned, additional factors seem to play a role for the gender differences observed.

The young adults aged 21 to 30 years constituted 51% of the study population and were more likely to receive the vaccine (p<0.041). This group is highly steered, by social media in Uganda [34], which contributes to rapid data sharing among the peers. The data however, cannot be generalized to the country because other studies have reported high vaccine uptake in older age groups [35, 36]. Nevertheless, it is encouraging to report that the majority group displayed a positive perception towards COVID-19 vaccination.

Sixty-three percent of the population had attained secondary education and 50% on average were self-employed. The level of education did not differ in individuals who accepted and those who did not accept the vaccine. Similarly, in resource-advantaged countries, high education level was not exclusively linked to better vaccine acceptance [37]. Those with salaried and self-employed jobs were more likely to accept the vaccine. This group of people are moderately exposed to stable jobs and tend to be less vulnerable a factor which has been linked a better COVID-19 vaccine uptake [38].

Married participants were more likely to be vaccinated. Couples have been reported to be less vulnerable as well and demonstrated an improved COVID-19 vaccine uptake when compared to divorced and separated participants [39]. The study, however, was performed among participants of an older age group. Acceptance of COVID-19 vaccines by Christians and Moslems did not differ and was not statistically significant. Thus, it would not give a valid comparison to other studies. Other studies have linked Christian patriotism and Islamic faith to reduced COVID-19 vaccine uptake [40, 41].

Thankfully, Uganda has been endowed with good political will and the President of the Republic of Uganda fostered COVID-19 vaccine development within the country [42]. The study was conducted in a peri-urban community at a time when the public had just been invited to receive the vaccine after vaccinating the priority high-risk groups and hence, the 1^st^ dose of COVID-19 vaccine receipt has been considered. The ‘studied practices’ hardly influenced the participants’ choices to take up the COVID-19 vaccine. The practices included willingness to accept and pay for the vaccine; and recommending COVID-19 vaccination to the close social support groups.

### Limitations

The study was conducted during early vaccine roll out and thus, the index vaccine uptake could not be predicted accurately. Secondly, it was a cross-sectional study and evaluation of the second dose of COVID-19 vaccine uptake was harder to estimate than the first dose. Since some participants had come to attend the clinic, these are likely to have a health care seeking behavior which might bias the results. This, however, would depend on the size of the group in question. The study was furthermore performed in a peri-urban setting and the results obtained would only be inferred to similar settings in the country.

## Conclusion and recommendation

Overall, COVID-19 vaccine uptake was low in the study population. This was attributed to negative beliefs in addition to the information gap on vaccine safety and related side effects. Sensitization campaigns that simultaneously address vaccine safety need to be considered to improve vaccine uptake in future.

## Supporting information

S1 Appendix (DOCX)
