# Supplementary material for: COVID-19 vaccine uptake and associated factors among individuals living in a peri-urban area in Uganda: A cross-sectional study
Source: PLoS One. 2024 Nov 4;19(11):e0312377. doi: 10.1371/journal.pone.0312377 (PMC11534251; doi:10.1371/journal.pone.0312377)
Supplement: S1 Appendix — (DOCX) [file pone.0312377.s001.docx]

**ANNEX**

| **COVID-19 VACCINE KAPC QUESTIONNAIRE**  **Practices and Concerns regarding the COVID-9 vaccine**  Please read the given questions/statements carefully and respond to the best of your knowledge: | | | | | | |
| --- | --- | --- | --- | --- | --- | --- |
| 20. It is legally mandatory to take COVID-19 vaccine   1. Yes 2. No 3. Don’t know | | | | | | |
| 1. Do you believe that vaccines can protect you from serious diseases?   Strongly disagree.  Disagree  Somewhat disagree.  Neither agree nor disagree  Somewhat agree.  Agree  Strongly agree. | | | | | | |
| 1. We have mentioned a group of people who may or may not be eligible for taking COVID-19 vaccine. Please mark your opinion for the same by checking the most appropriate option: | | | | | | |
| Group | Eligible | | | Not eligible | Don’t know | |
| (22.1) Infant <1 years of age |  | | |  |  | |
| (22.2) Children and adolescents <18 years of age |  | | |  |  | |
| (22.3) Adults ≥18 years |  | | |  |  | |
| (22.4) Pregnant ladies and lactating mothers |  | | |  |  | |
| (22.5) Patients with chronic diseases like diabetes, hypertension and heart diseases. |  | | |  |  | |
| (22.6) Persons having active COVID-19 infection |  | | |  |  | |
| (22.7) Persons recovered from COVID-19 infection |  | | |  |  | |
| (22.8) Persons allergic to food items/drugs |  | | |  |  | |
| (22.9) Immuno-compromised patients |  | | |  |  | |
| 1. Protective immunity against COVID-19 infection will be achieved after: 2. First dose of vaccination 3. Second dose of vaccination 4. Fourteen days after first dose of vaccination 5. Fourteen days after second dose of vaccination 6. Don’t know | | | | | | |
| 1. In the present era, there are multiple sources of information regarding a particular issue. **How significantly the following sources of information have influenced your opinion regarding vaccination?** | | | | | | |
| Source of information | Insignificant effect | | | Somewhat significant effect | Very significant effect | |
| (24.1) News from national TV/Radio |  | | |  |  | |
| (24.2) Government agencies e.g., MOH, political leaders… |  | | |  |  | |
| (24.3) Social media (Facebook, Instagram and WhatsApp) |  | | |  |  | |
| (24.4) Discussion amongst friends and family |  | | |  |  | |
| (24.5) Healthcare provider |  | | |  |  | |
| (24.6) Religious leaders |  | | |  |  | |
| (24.6) If there is any other source of information: Please specify: | | | | | | |
| From question 26-34, there are certain statements regarding different aspects of COVID-19 vaccination. Please mark the response which best explains your opinion regarding a particular statement: | | | | | | |
| 1. When my turn of vaccination comes, I am willing to take the COVID-19 vaccine. 2. Strongly agree 3. Agree 4. Neither agree nor disagree 5. Disagree 6. Strongly disagree | | | | | | |
| 1. I will prefer to acquire immunity against COVID-19 naturally (by having the disease/subclinical infection) rather than by vaccination.   (i) Strongly agree  (ii) Agree  (iii) Neither agree nor disagree  (iv) Disagree  (v) Strongly disagree | | | | | | |
| 1. I am willing to get the COVID-19 vaccine, even if I must pay to get it.   (i) Strongly agree  (ii) Agree  (iii) Neither agree nor disagree  (iv) Disagree  (v) Strongly disagree | | | | | | |
| 1. I will recommend my family and friends to get vaccinated against COVID-19.   (i) Strongly agree  (ii) Agree  (iii) Neither agree nor disagree  (iv) Disagree  (v) Strongly disagree | | | | | | |
| 1. If you have taken the vaccine, certain factors must have motivated you to do so. If you are waiting for your turn to get vaccinated, then certain factors might be responsible for your decision to take the vaccine. Given below, there are certain statements regarding this. Please mark your response which according to you best explains your opinion for each statement, respectively. | | | | | | |
| I have taken/will take the COVID-19 vaccine because | | Strongly agree | Disagree | Neither agree  nor disagree | Agree | Strongly agree |
| (29.1) I think there is no harm in taking COVID-19 vaccine | |  |  |  |  |  |
| (29.2) I believe COVID-19 vaccine will be useful in protecting me from the COVID-19 infection | |  |  |  |  |  |
| (29.3) COVID-19 vaccine is available free of cost. | |  |  |  |  |  |
| (29.4) My healthcare professional/doctor has recommended me. | |  |  |  |  |  |
| (29.5) I feel the benefits of taking the COVID-19 vaccine outweighs the risks involved. | |  |  |  |  |  |
| (29.6) I believe that taking a COVID-19 vaccine is a societal responsibility. | |  |  |  |  |  |
| (29.7) There is sufficient data regarding the vaccine’s safety and efficacy released by the government. | |  |  |  |  |  |
| (29.8) Many people are taking the COVID-19 vaccine. | |  |  |  |  |  |
| (29.9) I think it will help in eradicating COVID-19 infection. | |  |  |  |  |  |
| (30.10) My role models/political leaders/senior doctors/scientists have taken COVID-19 vaccine | |  |  |  |  |  |
| 1. There are still several concerns regarding the COVID-19 vaccine that may influence your decision (creating doubt in your mind) to get COVID-19 vaccine. Give your opinion on how the following statements have influenced/ will influence your decision to take the COVID-19 vaccine. | | | | | | |
| I am concerned that: | | Strongly agree | Disagree | Neither agree nor disagree | Agree | Strongly agree |
| (30.1) COVID-19 vaccine might not be easily available to me. | |  |  |  |  |  |
| (30.2) I might have immediate serious side effects after taking COVID-19 vaccine. | |  |  |  |  |  |
| (30.3) COVID-19 vaccine may be faulty or fake. | |  |  |  |  |  |
| (30.4) COVID-19 vaccine was rapidly developed and approved. | |  |  |  |  |  |
| (30.5) I might have some unforeseen future effects of the COVID-19 vaccine. | |  |  |  |  |  |
| (30.6) COVID-19 vaccine is being promoted for commercial gains of pharmaceutical companies. | |  |  |  |  |  |
| 1. After getting COVID-19 vaccine, I don’t need to follow preventative measures such as wearing a mask, sanitization, and social distancing.   (i) Strongly agree  (ii) Agree  (iii) Neither agree nor disagree  (iv) Disagree  (v) Strongly disagree | | | | | | |

| 1. If you have taken the vaccine, certain factors must have motivated you to do so. If you are waiting for your turn to get vaccinated, then certain factors might be responsible for your decision to take the vaccine. Given below, there are certain statements regarding this. Please mark your response which according to you best explains your opinion for each statement, respectively. | | | | | |
| --- | --- | --- | --- | --- | --- |
| I have taken/will take the COVID-19 vaccine because | Strongly agree | Disagree | Neither agree  nor disagree | Agree | Strongly agree |
| (30.1) I think there is no harm in taking COVID-19 vaccine |  |  |  |  |  |
| (30.2) I believe COVID-19 vaccine will be useful in protecting me from the COVID-19 infection |  |  |  |  |  |
| (30.3) COVID-19 vaccine is available free of cost. |  |  |  |  |  |
| (30.4) My healthcare professional/doctor has recommended me. |  |  |  |  |  |
| (30.5) I feel the benefits of taking the COVID-19 vaccine outweighs the risks involved. |  |  |  |  |  |
| (30.6) I believe that taking a COVID-19 vaccine is a societal responsibility. |  |  |  |  |  |
| (30.7) There is sufficient data regarding the vaccine’s safety and efficacy released by the government. |  |  |  |  |  |
| (30.8) Many people are taking the COVID-19 vaccine. |  |  |  |  |  |
| (30.9) I think it will help in eradicating COVID-19 infection. |  |  |  |  |  |
| (30.10) My role models/political leaders/senior doctors/scientists have taken COVID-19 vaccine |  |  |  |  |  |
| 1. There are still several concerns regarding the COVID-19 vaccine that may influence your decision (creating doubt in your mind) to get COVID-19 vaccine. Give your opinion on how the following statements have influenced/ will influence your decision to take the COVID-19 vaccine. | | | | | |
| I am concerned that: | Strongly agree | Disagree | Neither agree nor disagree | Agree | Strongly agree |
| (31.1) COVID-19 vaccine might not be easily available to me. |  |  |  |  |  |
| (31.2) I might have immediate serious side effects after taking COVID-19 vaccine. |  |  |  |  |  |
| (31.3) COVID-19 vaccine may be faulty or fake. |  |  |  |  |  |
| (31.4) COVID-19 vaccine was rapidly developed and approved. |  |  |  |  |  |
| (31.5) I might have some unforeseen future effects of the COVID-19 vaccine. |  |  |  |  |  |
| (31.6) COVID-19 vaccine is being promoted for commercial gains of pharmaceutical companies. |  |  |  |  |  |
| 1. After getting COVID-19 vaccine, I don’t need to follow preventative measures such as wearing a mask, sanitization, and social distancing.   (i) Strongly agree  (ii) Agree  (iii) Neither agree nor disagree  (iv) Disagree  (v) Strongly disagree | | | | | |
